# Age-Dependent Decrease in the Induction of Regulatory T Cells Is Associated With Decreased Expression of RALDH2 in Mesenteric Lymph Node Dendritic Cells

**DOI:** 10.3389/fimmu.2020.01555

**Published:** 2020-08-11

**Authors:** Tomohiro Takano, Ryutaro Kotaki, Jihyun Park, Tadashi Yoshida, Yoshio Wakatsuki, Masaru Tanokura, Takuya Miyakawa, Kyoko Takahashi, Haruyo Nakajima-Adachi, Satoshi Hachimura

**Affiliations:** ^1^Research Center for Food Safety, The University of Tokyo, Tokyo, Japan; ^2^Department of Applied Biological Chemistry, The University of Tokyo, Tokyo, Japan; ^3^Department of Applied Biological Science, Tokyo University of Agriculture and Technology, Tokyo, Japan; ^4^Department of Clinical Bio-Regulatory Science, Kyoto University Graduate School of Medicine, Kyoto, Japan; ^5^Department of Applied Biological Science, College of Bioresource Sciences, Nihon University, Kanagawa, Japan

**Keywords:** aging, regulatory T cells, dendritic cells, RALDH2, retinoic acid, epigenetic regulation

## Abstract

A decline in immune function with aging has been reported. Regulatory T cell (Treg) induction is known to decrease with age, and elucidating the underlying mechanism is important for preventing age-related diseases due to age-related chronic inflammation. In the intestine, dendritic cells (DCs) play an important role in inducing Tregs specific to oral antigens, and they efficiently induce Tregs via production of retinoic acid (RA), a vitamin A metabolite, catalyzed by the enzyme retinaldehyde dehydrogenase 2 (RALDH2). We have previously reported that in the mesenteric lymph node (MLN), a secondary lymphoid tissue in which immune responses to oral antigens are induced, four DC subsets express different levels of CD11b, CD103, and PD-L1, and we have reported that the CD11b^–^CD103^+^PD-L1^high^ subset expresses the highest levels of the RALDH2 gene and induces Tregs *in vitro*. We examined Treg induction in young and aged mice using a Treg induction model by administering a food antigen, and we found that antigen-specific Treg induction was decreased in aged mice. We further investigated the MLN DCs, and a significant decrease in RALDH2 gene expression was observed in MLN DCs from aged mice. As factors, we found that the proportion of the CD11b^–^CD103^+^PD-L1^high^ subset was decreased in aged mice compared with that in young mice and that RALDH enzyme activity was decreased in the CD11b^–^CD103^+^PD-L1^high^ and CD11b^+^CD103^+^PD-L1^high^ subsets. Furthermore, analysis of the methylation of the RALDH2 gene promoter region revealed that CpG motifs were more methylated in the MLN DCs of aged mice, suggesting that RALDH2 expression was suppressed by epigenetic changes. Finally, we found that RA treatment tended to increase Treg induction. These results suggest that the regulation of RA production may be involved in the age-related decrease in antigen-specific Treg induction.

## Introduction

Over decades, numerous studies have revealed that aging is involved in defects in immunological functions, and a relationship between aging and chronic inflammatory conditions has been reported (1). Age-related low-grade chronic inflammation, called inflamm-aging, is known for its elevated inflammatory cytokines, such as interleukin (IL)-6, IL-1β, and tumor necrosis factor (TNF)-α in serum (2–4), and it is considered a risk factor that triggers age-related diseases such as heart diseases or cerebral infarction (5). Understanding the mechanism underlying inflamm-aging is important for maintaining health in the elderly and preventing aging-related diseases.

Immunosuppressive function is precisely controlled to avoid an excessive immune response to harmless substances in mucosal tissues such as intestinal tracts which are exposed to a large amount of antigens. Uncontrolled immunosuppressive function can lead to acute or chronic inflammation such as inflammatory bowel diseases (IBD). Impairment of the induction of the immunosuppressive response may be involved in age-related inflammation.

The regulatory T cell (Treg) is one of major cell types responsible for immunosuppressive function in the intestinal immune system (6, 7). Tregs can be divided into two types: thymus-derived Tregs (tTregs) that differentiate in the thymus, and peripherally derived Tregs (pTregs) that are induced in peripheral tissues (8). It has been reported that the number of tTregs increases in blood, lymphoid tissues, and peripheral tissues with aging (9). Conversely, some *in vivo* and *in vitro* experiments have shown that pTreg induction decreases with aging (10–12). Although the clear link between inflamm-aging and Tregs has not been demonstrated, it is attractive to assume that reduced induction of pTregs is associated with a failure to suppress age-related inflammation.

Dendritic cells (DCs) are Treg inducer cells, and intestinal DCs are known to have a high ability to induce Tregs with production of retinoic acid (RA), the active vitamin A metabolite, and transforming growth factor (TGF)-β (13). Intestinal DCs have been reported to have high expression of retinal dehydrogenase 2 (RALDH2), which is involved in the synthesis of RA (14), and integrin αvβ8, which is involved in the conversion of latent TGF-β to active TGF-β (15, 16). In our previous study, it was found that DCs in the mesenteric lymph node (MLN) consist of four subsets, expressing different levels of CD11b, CD103, and PD-L1 with distinct functions, and it was revealed that the CD11b^–^CD103^+^PD-L1^high^ population has an especially high ability to induce Tregs (17).

To examine the influence of aging on the induction of pTregs, an experimental model that can artificially induce an antigen-specific reaction without being affected by tTreg accumulation is required. In our previous study, we reported that administration of a diet containing ovalbumin (OVA) to DO11.10 mice expressing OVA-specific T cell receptors could induce the antigen-specific immune response in the intestinal immune system. In this model, a 7-day administration of the OVA diet induced Tregs in the MLN and established oral tolerance (18). In this study, we used RAG2KO/DO11.10 mice lacking recombination activating gene (RAG) to obtain mice with only OVA-specific T cells and very little if any pTregs. By aging these mice and administering the OVA-containing diet, it is possible to evaluate the accurate impact of aging on antigen-specific Treg induction.

## Materials and Methods

### Animals

BALB/c mice were purchased from Charles River Laboratories Japan (Yokohama, Japan). DO11.10 mice (19) and RAG2KO/DO11.10 mice (DO11.10 mice deficient in recombination-activating gene 2) on a BALB/c background were maintained and obtained from Sankyo Labo Service (Tokyo, Japan). These mice were fed CE-2 diet (CLEA Japan, Shizuoka, Japan) and distilled deionized water *ad libitum* and maintained in a specific pathogen-free environment in a temperature-controlled room, using a 12-h light-dark cycle. Eight to twelve-week-old mice were used as young mice, and 18–27-month-old mice were used as aged mice. Gender-matched mice were used. All experiments were performed in accordance with the guidelines for the care and use of laboratory animals of the University of Tokyo.

### Induction of Tregs in MLNs

The control casein (CN) diet and the egg-white (EW) diet were prepared as described previously (20). For induction of Tregs, RAG2KO/DO11.10 mice were fed the EW diet for 7 days *ad libitum*, and the control group mice were fed CN diet for the same period. To evaluate the effects of RA administration on Treg induction, 300 μg all-trans retinoic acid (FUJIFILM Wako Pure Chemical Corporation, Osaka, Japan) dissolved in 50 μl dimethyl sulfoxide (DMSO) (Sigma-Aldrich, St Louis, MO, United States) or DMSO was intraperitoneally injected and repeated daily for 7 days.

### Media and Reagents

RPMI 1640 (Nissui Pharmaceutical, Tokyo, Japan) containing 100 U/ml penicillin G potassium (Meiji Seika Pharma, Tokyo, Japan), 100 μg/ml streptomycin sulfate (Meiji Seika Pharma), 50 μM 2-mercaptoethanol (Tokyo Chemical Industry, Tokyo, Japan), 0.03% l-glutamine (FUJIFILM Wako Pure Chemical Corporation), and 0.2% sodium hydrogen carbonate (FUJIFILM Wako Pure Chemical Corporation) was prepared with 10% heat-inactivated fetal calf serum. For flow cytometry, anti-CD4-APC (GK1.5), anti-CD11c-APC/Cy7 (N418), purified anti-CD16/32 (93), anti-CD103-biotin (2E7), anti-CD274 (PD-L1)-PE (10F.9G2), and streptavidin-PE/Cy7 were purchased from BioLegend (San Diego, CA, United States); anti-Foxp3-PE (FJK-16s) was purchased from eBioscience (San Diego, CA, United States); and anti-CD11b-APC (M1/70) was purchased from TONBO biosciences (San Diego, CA, United States). DMSO was purchased from Sigma-Aldrich, and all-trans-retinoic acid (RA) was purchased from FUJIFILM Wako Pure Chemical Corporation. OVA323-339 peptide (OVAp) (ISQAVHAAHAEINEAGR) was purchased from Operon Biotechnologies (Tokyo, Japan).

### Immune Cell Preparation

For DC isolation, MLNs obtained from BALB/c mice were digested in 10% FCS-RPMI containing 0.5 mg/ml collagenase (FUJIFILM Wako Pure Chemical Corporation) with 10 μg/ml DNase I (Roche Diagnostics GmbH, Mannheim, Germany), and a cell suspension was obtained by filtering the digestion. From the obtained whole cells, CD11c^+^ cells were magnetically purified using the magnetic activated cell sorting (MACS) cell separation system (Miltenyi Biotec, Bergisch Gladbach, Germany) according to the manufacturer’s instructions. To obtain CD11c^+^ cells for culture, CD11c^+^ purification with MACS was conducted twice to achieve high purity, and CD11c^+^ cells were used as DCs. For CD4^+^ T cell isolation, splenocytes were obtained from RAG2KO/DO11.10 mice. From whole splenocytes, CD4^+^ cells were separated with the MACS system. Cells were isolated from multiple mice and pooled to obtain the required number of cells. For analysis of CpG motifs methylation, MACS-enriched MLN CD11c^+^ cells were sorted by FACS Aria II (BD Bioscience, Franklin Lakes, NJ, United States). PBS containing 2% FCS was used as staining and washing buffer.

### Cell Culture

To analyze Foxp3 expression, MLN DCs (2 × 10^4^ cells) from BALB/c mice and splenic CD4^+^ cells (2 × 10^5^ cells) from RAG2KO/DO11.10 mice were cocultured in the presence of OVAp (100 nM) in a 96-well flat bottom plate (Corning, New York, NY, United States) in 200 μl of 10% FCS-RPMI for 72 h in a 5% CO_2_ humidified atmosphere at 37°C.

### qPCR

Total RNA was extracted from the cells using QIAShredder (QIAGEN, Hilden, Germany) and the RNeasy Mini Kit (QIAGEN). cDNA was synthesized with SuperScript VILO MasterMix (Thermo Fisher Sciences, Waltham, MA, United States). Subsequently, real-time PCR was performed to measure the relative gene expression with the QuantiTect SYBR Green PCR Kit (QIAGEN) and LightCycler (Roche Diagnostics GmbH). The relative gene expression was calculated assuming that the targeted cDNA was doubled at one cycle. The results were normalized to Gapdh gene expression as the internal control. The following primers were used: 5′-TGTCCGTCGTGGATCTGAC-3′, forward, and 5′-CCTGCTTCACCACCTTCTTG-3′, reverse, for *Gapdh*; 5′-GACTTGTAGCAGCTGTCTTCACT-3′, forward, and 5′-TCACCCATTTCTCTCCCATTTCC-3′, reverse, for *Aldh1a2*.

### Flow Cytometry

The cells were stained with fluorescently labeled antibodies after Fc receptor blockade by anti-CD16/32 antibody. When biotinylated antibodies were used in the staining, secondary staining was performed using streptavidin-conjugated fluorescent reagents after washing away the primary staining antibodies. To stain dead cells with propidium iodide, propidium iodide (2 μg/ml; Sigma-Aldrich) was added to samples after staining with antibodies and washed out immediately. Intracellular Foxp3 was stained using Fixation/Permeabilization Concentrate, Diluent, and Permeabilization buffer (eBioscience) according to the manufacturer’s instructions. To evaluate RALDH activity, the ALDEFLUOR assay kit (StemCell Technologies, Vancouver, Canada) was used, and RALDH activity was confirmed by comparing the control sample containing the ALDH inhibitor (N,N-diethylaminobenzaldehyde; DEAB). The fluorescence levels were measured by FACS Verse (BD Bioscience) and data analyzed using FLOWJO software (BD).

### Bisulfite Sequencing

Genomic DNA was prepared from sorted cells using a PureLink Genomic DNA Mini Kit (Invitrogen, Carlsbad, CA, United States) according to the manufacturer’s instructions. To analyze the methylation of CpG motifs, purified genomic DNA was modified by the bisulfite conversion reaction using an EpiMark Bisulfite Conversion Kit (New England BioLabs, Ipswich, MA, United States). The modified DNA was subjected to PCR using TaKaRa EpiTaq^TM^ HS (for bisulfite-treated DNA) (Takara Bio, Shiga, Japan). The RALDH2 promoter region (−389 to +5 bp) was amplified by PCR from modified genomic DNA. The following primers were used: 5′-ATTTGGAATATTTAGGTAATTT-3′, forward, and 5′-ACTATATATAAACAAATATCAAA-3′, reverse, for 1st PCR; 5′-GAGTATTTATTATTTTATTTAG-3′, forward, and 5′-ACTATATATAAACAAATATCAAA-3′, reverse, for nested PCR. After amplification, the PCR products were obtained using gel electrophoresis and purified using a MinElute Gel Extraction Kit (QIAGEN). The purified PCR products were cloned into the pCR2.1 vector for sequencing. Nucleotide sequences were analyzed using Big Dye Terminator on an ABI3130xl sequencer (Applied Biosystems, Foster City, CA, United States). The methylation state for each CpG site in the PCR products was analyzed using a web-based freely available quantification tool for methylation analysis (QUMA)^1^.

### Statistical Analysis

Results are shown as the mean ± SD. Student’s *t*-test, Dunnett’s test, or Tukey’s HSD test were used for statistical analyses. Differences were considered significant at *p* < 0.05.

## Results

### Induction of Antigen-Specific Tregs Was Decreased in Aged Mice

**FIGURE 1:**
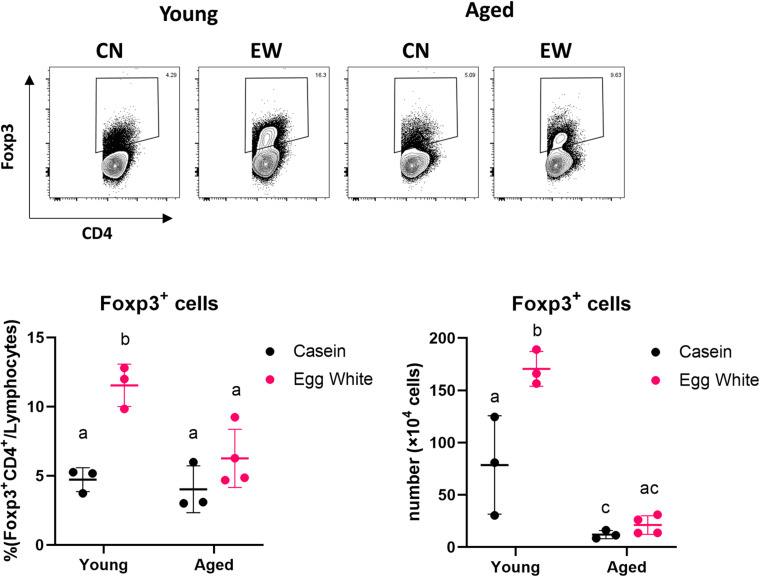
Impact of aging on antigen-specific Treg induction. The proportion and absolute number of CD4^+^Foxp3^+^ cells in MLNs from young and aged RAG2KO/DO11.10 mice was analyzed. The gating strategy used to identify CD4^+^Foxp3^+^ cells is shown in Supplementary Figure S1. Data are shown as the mean ± SD of independent mice (*n* = 3–4), and each point indicates the data from independent mice. The plot shows one representative data of two experiments. Tukey’s HSD test was used for statistical analysis. Values not sharing a common letter are significantly different (*p* < 0.05).

We used the oral tolerance model to examine the changes in the induction of Tregs with aging. We have previously reported that feeding an ovalbumin-containing diet (EW diet; egg-white diet) to DO11.10 mice for 7 days induced OVA-specific Tregs in MLNs (18). We fed the EW diet for 7 days to young or aged RAG2KO/DO11.10 mice, which have OVA-specific T cells. We found that the proportion and numbers of CD4^+^Foxp3^+^ Tregs induced in aged mouse MLNs was significantly lower than young mice (Figure 1). This result suggested that Treg-inducing function decreased with age.

### Gene Expression of RALDH2 in MLN DCs Was Decreased in Aged Mice

**FIGURE 2:**
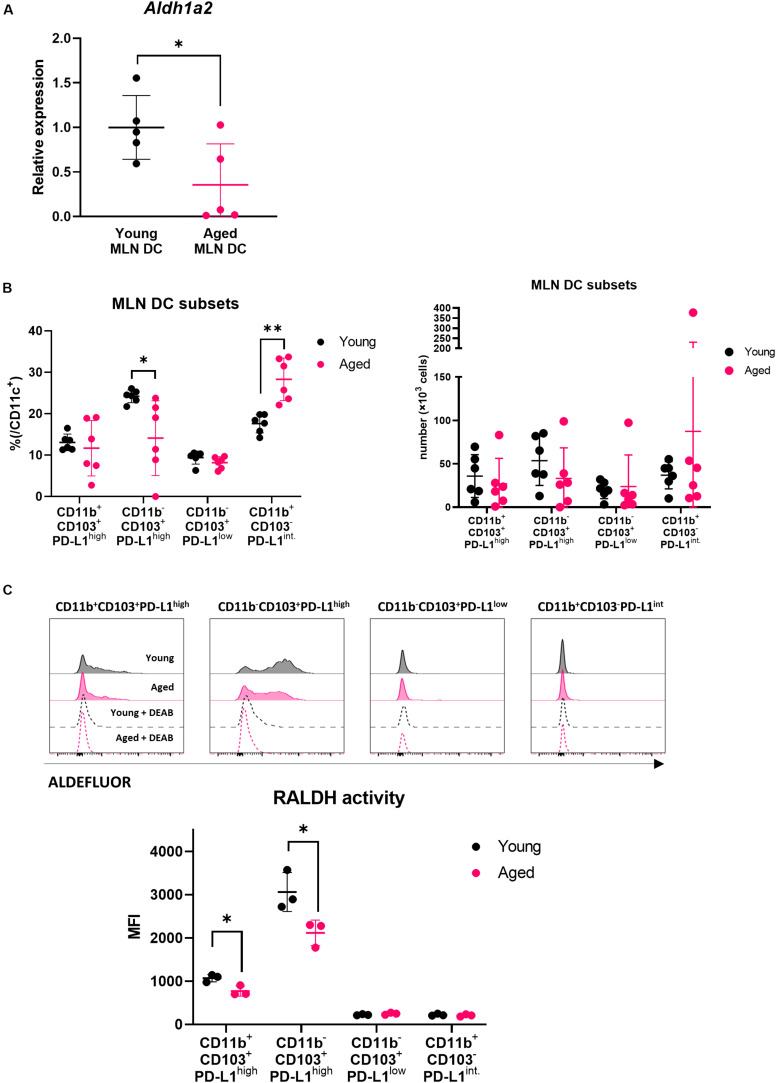
Impact of aging on MLN DCs. **(A)** The relative gene expression of *Aldh1a2* in MLN DCs of young and aged BALB/c mice was measured. Data are shown as the mean ± SD of five independent experiments, and each point indicates the data from independent experiments. Student’s *t*-test was used for statistical analysis (*p < 0.05). **(B)** The proportion and absolute number of MLN DC subsets in young and aged BALB/c mice was analyzed. The gating strategy used to identify MLN DC subsets is shown in Supplementary Figure S2A. Data are shown as the mean ± SD of independent mice (*n* = 6), and each point indicates the data from independent mice. The plot shows one representative dataset of two independent experiments. Student’s *t*-test was used for statistical analysis (*p < 0.05, **p < 0,01). **(C)** RALDH activity of MLN DC subsets from young and aged BALB/c mice was analyzed by flow cytometry. The gating strategy used to identify MLN DC subsets is shown in Supplementary Figure S2A. Histogram plots indicate the RALDH activity in each MLN DC subsets measured using ALDEFLUOR reagent (solid black line: young MLN DC, solid magenta line: aged MLN DC, dashed black line: young MLN DC + DEAB, dashed magenta line: aged MLN DC + DEAB). Data are shown as the mean ± SD of independent mice (*n* = 3), and each point indicates the data from independent mice. The plot shows one representative dataset from two independent experiments. Student’s *t*-test was used for statistical analysis (*p < 0.05).

Several reports have shown that DCs showing retinaldehyde dehydrogenase (RALDH) activity promote Treg differentiation through production of RA (21–23). We have previously revealed that DCs in aged intestinal lymphoid organs express lower levels of the RALDH gene (24). MLN DCs (CD11c^+^ cells) were purified from young and aged BALB/c mice, and real-time PCR was performed to examine the gene expression. It was confirmed that the gene expression of RALDH2 was significantly reduced in aged mice (Figure 2A).

### The Proportion and RALDH Activity of MLN DC Subsets Altered With Aging

It has been reported that MLN DCs are a heterogenous population expressing different levels of phenotypic markers (21). Our previous study revealed that MLN DCs are divided into four subsets (CD11b^+^CD103^+^PD-L1^high^, CD11b^–^CD103^+^PD-L1^high^, CD11b^–^CD103^+^PD-L1^low^, and CD11b^+^CD103^–^PD-L1^int.^). The CD11b^–^CD103^+^PD-L1^high^ subset shows the highest expression of the RALDH2 gene (*Aldh1a2*) and induces Treg most efficiently *in vitro* (17).

We hypothesized that the reduction of RALDH2 gene expression with aging was related to the changes in MLN DC subset composition. We analyzed the ratio of the subsets of MLN DCs in young or aged mice by flow cytometry. The CD11b^–^CD103^+^PD-L1^high^ subset, which had the highest RALDH2 activity, was significantly decreased in aged mice compared with young mice, and instead, the CD11b^+^CD103^–^PD-L1^int^ subset increased (Figure 2B). There was no significant difference concerning the numbers of DC subsets between young and aged mice, although there appeared to be a tendency of decrease in the CD11b^–^CD103^+^PD-L1^high^ subset in aged mice. Next, we examined RALDH activity of the four MLN DC subsets by flow cytometry to confirm the correlation between the gene expression of RALDH2 and the enzymatic activity. In accordance with the gene expression, the CD11b^–^CD103^+^PD-L1^high^ subset had the highest RALDH2 activity, followed by the CD11b^+^CD103^+^PD-L1^high^ subset. The CD11b^–^CD103^+^PD-L1^low^ and CD11b^+^CD103^–^PD-L1^int.^ subsets had low or intermediate RALDH2 activity (Supplementary Figure S2B).

We compared the RALDH activity in the MLN DC subsets in young and aged mice by flow cytometry, and we found that RALDH enzyme activity was reduced in the CD11b^–^CD103^+^PD-L1^high^ and CD11b^+^CD103^+^PD-L1^high^ subsets (Figure 2C). These results suggested that the changes in MLN DC subset composition and RALDH enzyme activity were related to the reduction of RALDH2 gene expression.

### Impact of Aging on MLN DCs in Inducing Foxp3 Expression

**FIGURE 3:**
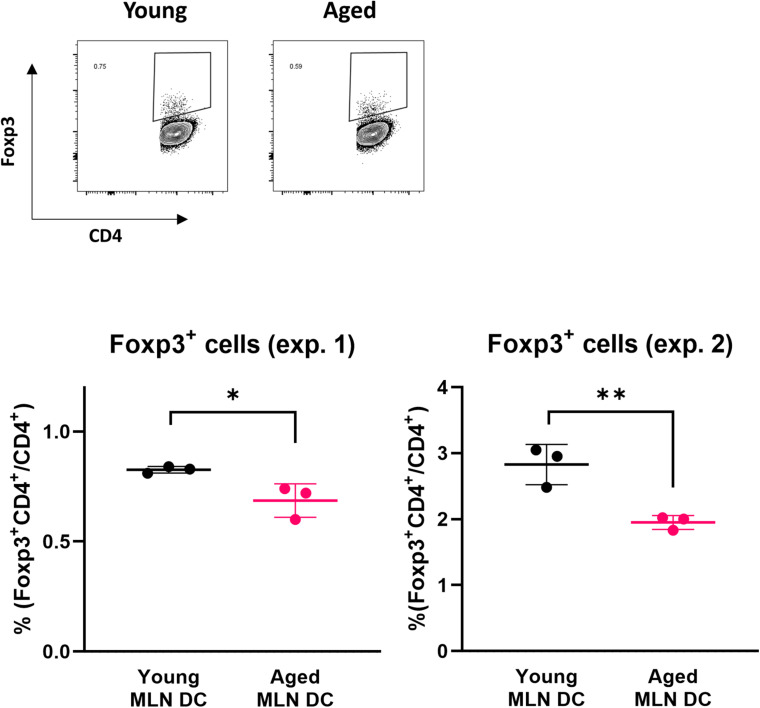
Impact of aging on Foxp3 expression in a DC-T cell coculture. The proportion of Foxp3^+^ CD4^+^ cells after coculture of spleen CD4^+^ T cells from RAG2KO/DO11.10 mice and MLN DCs from young or aged BALB/c mice was analyzed. The gating strategy used to identify CD4^+^Foxp3^+^ cells is shown in Supplementary Figure S3. Data are shown as the mean ± SD of independent wells (*n* = 3), and each point indicates the data from independent wells. The plots show datasets from two independent experiments. Student’s *t*-test was used for statistical analysis (*p < 0.05, **p < 0.01).

Retinoic acid production mediated by RALDH2 in DCs affects T cells to enhance the expression of Foxp3, a transcription factor that induces differentiation into Tregs. We examined whether the ability to induce Foxp3^+^ T cells was reduced in MLN DCs of aged mice using DC-T cell coculture systems. The expression level of Foxp3 in T cells was analyzed by flow cytometry after coculture of MLN DCs of young or aged BALB/c mice and CD4^+^ T cells derived from RAG2KO/DO11.10 mouse splenocytes. A decrease in Foxp3 induction was observed in the CD4^+^ T cells cocultured with MLN DCs from aged mice compared with young mice (Figure 3).

### Methylation of the RALDH2 Gene Promoter Region Was Enhanced in MLN DCs Derived From Aged Mice

**FIGURE 4:**
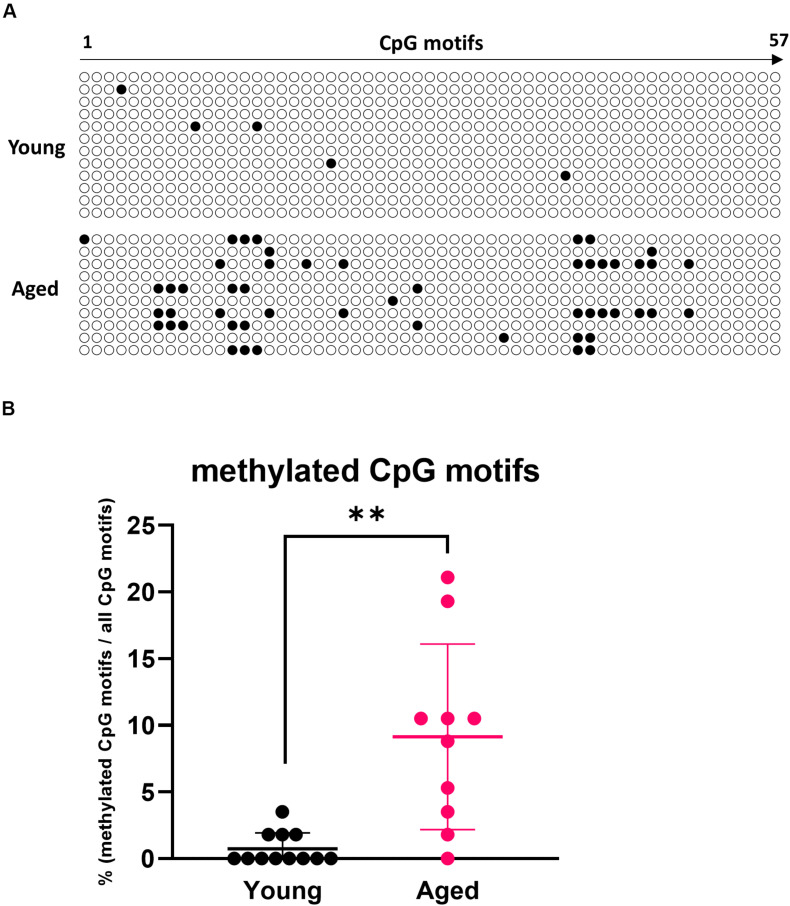
Analysis of methylation in CpG motifs of the MLN DC RALDH2 promoter region. **(A)** The methylation patterns of 57 CpG motifs in the RALDH2 promoter region in MLN DCs from young and aged BALB/c mice are shown. Closed circles indicate methylated CpG motifs, and open circles indicate unmethylated CpG motifs. Data for 12 and 10 clones obtained from young and aged mice, respectively, are shown. **(B)** The proportion of methylated CpG motifs in the RALDH2 gene promoter region were analyzed. Data are shown as the mean ± SD of 12 (young) and 10 (aged) independent clones, respectively, and each point indicates the data from independent clones. The plot shows one representative dataset from two independent experiments. Student’s *t*-test was used for statistical analysis (**p < 0.01).

Epigenetic changes such as methylation of CpG motifs are known as factors that suppress gene expression with aging (25). We measured the degree of methylation of CpG motifs in the region containing the RALDH2 gene promoter (−389 to +5 bp) (26) using bisulfite sequencing. It was revealed that CpG motifs in the RALDH2 gene promoter region of aged mouse MLN DCs were significantly methylated compared with young mice (Figure 4).

### Effects of RA Administration on Treg Induction

**FIGURE 5:**
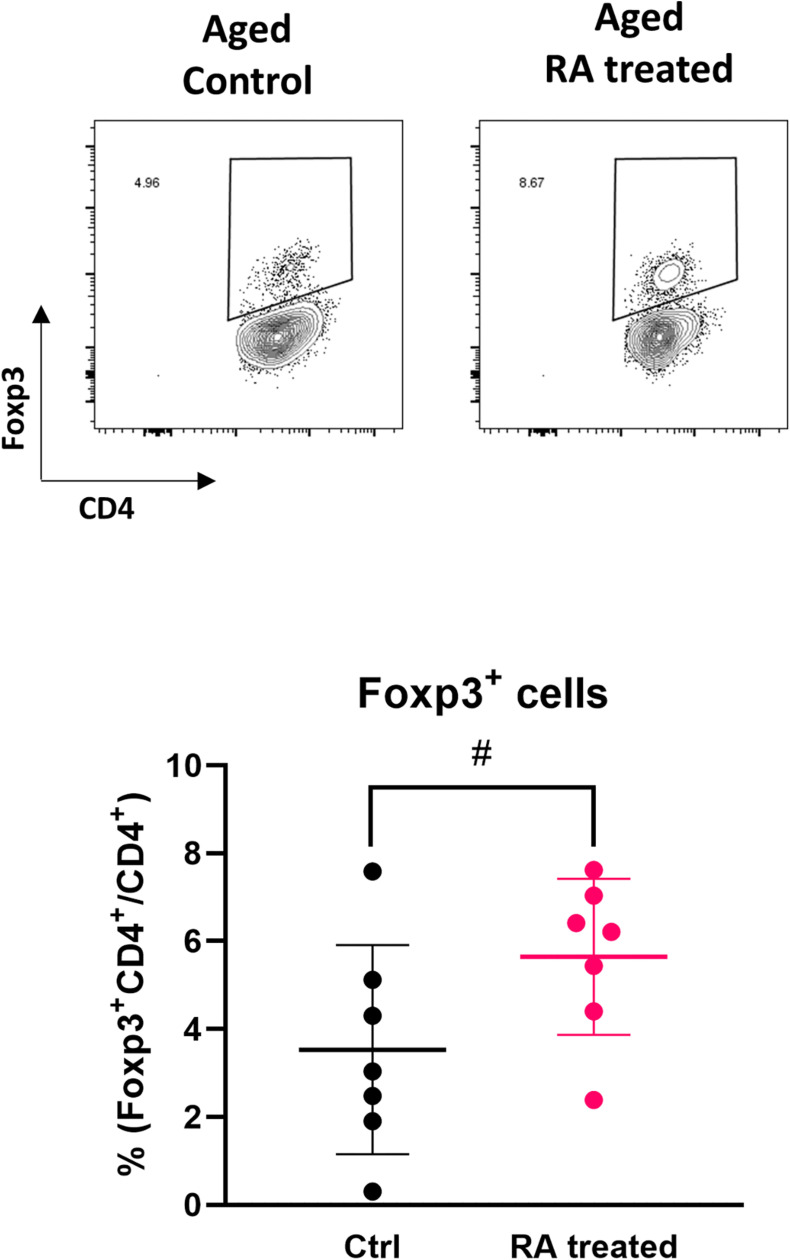
Effect of RA administration on the induction of antigen-specific Tregs. The proportion of CD4^+^Foxp3^+^ cells in MLNs were analyzed. The gating strategy used to identify CD4^+^Foxp3^+^ cells is shown in Supplementary Figure S4. Data are shown as the mean ± SD of independent mice (*n* = 7), and each point indicates the data from independent mice. Student’s *t*-test was used for statistical analysis (*#p* < 0.1).

To investigate whether the influence of aging on Treg induction by EW feeding was due to a decrease in RA production, RA was administered to aged RAG2KO/DO11.10 mice and the recovery of Treg induction was examined. RA was intraperitoneally administered once per day simultaneously with the start of EW diet feeding, and the proportion of Tregs in the MLN was measured 7 days later by flow cytometry. It was observed that Treg induction tended to increase with RA administration (Figure 5), which suggested that Treg induction was reduced in aged mice via a decrease in retinoic acid production.

## Discussion

In this study, we found aging-related alterations in MLN DCs. In aged mice, the MLN DC subsets which have high RALDH gene expression and enzymatic activity in young mice, decreased in its frequencies and RALDH activity. These age-related changes were reflected in decreased RALDH2 gene expression of overall MLN DC population. These results suggested that the ability to produce RA was decreased in the intestinal immune system of aged mice. In addition, methylation of the CpG motifs in the promoter region was increased in MLN DCs of aged mice, suggesting that RA production is regulated by epigenetic changes. To date, there have been no reports showing that decreased RALDH expression in the intestinal immune organs with aging is associated with Treg induction. Treg induced in the intestinal immune system controls the immune response to oral antigens. Our results suggest that aging leads to the decreased RA production by MLN DCs, which subsequently reduces the induction of Tregs and contributes to inflamm-aging.

Several groups have reported that the ability to induce Tregs decreases with age. According to Carpentier et al., a low rate of Treg differentiation was observed 28 days after the transfer of T cells from aged C57BL/6 mice to young mice (12). In addition, *in vitro* experiments showed that when T cells were cultured in the presence of IL-2 and TGF-β after stimulation with CD3 antibody, the rate of differentiation into Tregs of aged mice was lower compared with that of young mice (11, 12). Another study has reported that oral tolerance was established even in some strains at 8 weeks of age, but it was no longer established at 24 weeks of age (10). However, to our knowledge, no experimental system has evaluated Treg induction by an antigen-specific reaction in aged animals. In general, it is known that tTregs that differentiate from the thymus accumulate with aging (9, 27). In this study, by aging RAG2KO/DO11.10 mice and administering the OVA-containing diet, it is considered that Treg induction in aged mice can be observed without being affected by tTreg accumulation. Our results showed for the first time that Treg induction against new antigens was decreased in aged animals.

Numerous reports have already shown the effects of RA on the induction of Tregs (28–30). However, few reports have demonstrated that RA reduction is involved in the decrease in Treg induction with aging. A report by Bian et al. suggested that ALDH activity was reduced in conjunctiva cells of aged mice, which contributed to the inflammation that causes dry eye (31). Taken together with our results, it is suggested that the anti-inflammatory response is reduced in aged mice by a common process of RALDH reduction in mucosal immune systems. Although we focused on the intestinal immune system, considering that the Tregs induced in the intestine may circulate throughout the body, the decrease of Treg induction in the intestine may lead to systemic inflammation.

The significant decrease in the ratio and the tendency to decrease in the numbers of CD11b^–^CD103^+^PD-L1^high^ subset in aged mice should result in a decrease in the interaction of this DC subset with T cells, leading to decreased Foxp3^+^ T cell induction. We cannot deny the possibility that the MLN DC subset composition and RALDH activity changed as a result of a decrease in the ability to migrate from the lamina propria to MLNs in a subset that strongly expresses RALDH. In fact, the CD103^+^ subset of DCs has been reported to migrate to the MLN depending on CCR7 expression after acquiring antigen in the lamina propria (32, 33). The alterations in the migration properties of DC subsets with aging would be an interesting subject to examine in the future.

Epigenetic modification has been reported to regulate gene expression with aging (25). Hypermethylation of the CpG island region of the gene promoter region can lead to suppression of gene expression. In this report, we found that on average approximately 10% of CpG motifs in the RALDH2 gene promoter region of MLN DCs from aged mice were methylated, although MLN DCs from young mice were rarely methylated. Because the MLN DCs used in the analysis of methylation were not fractionated into subsets, it was not determined whether methylation of the promoter region occurred subset-specifically or non-specifically. Although Ohoka et al. have reported that methylation of the promoter region does not occur even in splenic DCs that do not normally express RALDH2 (26), it is possible that the expression of RALDH2 is suppressed in the specific subset by epigenetic regulation. Further analysis is needed to elucidate the specific factors that enhance methylation of CpG motifs with aging.

The decrease in Treg induction with aging can be caused not only by DC but also T cell functional decline. In fact, a decrease in immune function with aging has been reported in the acquired immune system (34). The decrease in Treg induction with aging observed in our experimental Treg induction model in response to oral antigens is likely to be due to the combined effects of DCs and T cells. To examine the effects of aging on T cells, we analyzed Treg induction *in vitro* using T cells isolated from young and aged RAG2KO/DO11.10 mice. T cells from aged mice had lower Foxp3 expression levels than T cells from young mice in the presence of TGF-β, a potent inducer of Tregs (Supplementary Figure S5). RA is a molecule that is involved in inducing T cell expression of C-C chemokine receptor type 9 (CCR9), a homing receptor for the intestinal tract, and inducing IgA production in B cells (14, 35). We further investigated the impact of aging on the expression of CCR9 *in vitro* and found that the enhanced induction of CCR9 dependent on RA was significantly decreased in T cells from aged mice (Supplementary Figure S6). These results suggest that T cell responses, possibly including sensitivity to TGF-β or RA, decrease with age. Decreased immune function with aging can induce impairment of the deployment of T cells in the intestinal tract and decreased IgA production through reduced RA production or responses to RA and can contribute to a decline in homeostasis.

Intestinal RALDH expression has been reported to depend on dietary vitamin A (36). The possibility that our observed decrease in RALDH expression might be related to a decrease in the amount of dietary vitamin A due to a decrease in food intake or digestive function accompanying aging cannot be ruled out. However, to produce vitamin A-deficient mice, it is necessary to administer a vitamin A-deficient diet for a long period (14). Considering that the normal diet used in this study contained a relatively high amount of vitamin A, a reduction in the diet is unlikely to be a direct cause.

It was difficult to investigate whether the alterations in DCs observed in this study affected inflamm-aging. Even if DCs derived from aged mice are transferred into young mice, it is considered very difficult to evaluate the effect of the transferred cells since the life span of DCs is several days (37). The production of chimeric mice by transferring bone marrow cells from aged mice may also be considered. However, examination of the RALDH activity in bone marrow-derived DCs induced from young and aged mice did not show a significant difference (data not shown). This result suggests that there is no difference in RA production at the time of development from bone marrow stem/progenitor cells. A different experimental system is needed to determine the precise role of the decrease in DC function in the dysregulation of the immune system caused by aging.

In the RA administration experiment, a tendency to promote Treg induction was observed, implying that the application of RA restored Treg induction in aged mice. Several studies have reported that RA administration has anti-inflammatory effects and enhances Treg induction. Bai et al. reported that intraperitoneal administration of RA ameliorates trinitrobenzene sulfonic acid (TNBS)-induced colitis and up-regulates Foxp3 expression in colonic tissues (38). Similar results have been shown in the DSS-induced colitis model (39). These data support the idea that RA administration could improve the age-related inflammation through restoring Treg induction.

In the present study, we showed a relationship between decreased Treg induction with age and alterations in MLN DC RA production. To date, no reports have clarified the underlying mechanism of the development of inflamm-aging. Several groups have proposed theories concerning the factors involved in age-related inflammation, including: oxidative stress, DNA damage, senescence in stem cells (40). However, the idea that dysregulation of immunosuppressive function triggers age-related inflammation has not been considered experimentally. We found that the induction of antigen-specific Tregs was reduced, and the decrease was associated with the decline in the function of DCs in aged mice. The results obtained from this study support the idea that a decrease in the control of the immune response by Tregs due to a decrease in the function of DCs may be a cause of the development of age-related chronic inflammation. Although further investigation is needed to reveal the underlying mechanism of inflamm-aging, clarifying the mechanism of the change in DCs with age may provide clues to combat age-related inflammation.

## Data Availability Statement

The raw data supporting the conclusions of this article will be made available by the authors, without undue reservation.

## Ethics Statement

The animal study was reviewed and approved by Institutional Animal Care and Use Committee, Graduate School of Agriculture and Life Sciences, The University of Tokyo.

## Author Contributions

TT designed the study, performed the experiments, analyzed the data, and wrote the manuscript. RK contributed to the dendritic cell experiments and reviewed the manuscript. JP and TY contributed to the dendritic cell experiments. YW provided the RAG2KO/DO11.10 mice. MT and TM contributed to experiments on aging and reviewed the manuscript. KT supervised the experiments on epigenetic modification. HN-A supervised the study and reviewed the manuscript. SH designed and supervised the study and critically edited the manuscript. All authors read and approved the manuscript.

## Conflict of Interest

The authors declare that the research was conducted in the absence of any commercial or financial relationships that could be construed as a potential conflict of interest.

## Footnotes

1 http://quma.cdb.riken.jp/top/index_j.html

## Supplementary Material

FIGURE S1 Gating strategy used to identify CD4^+^Foxp3^+^ cells. CD4^+^ cells were gated from FSC-SSC gated cells, and the proportion of Foxp3^+^CD4^+^ cells were analyzed.

FIGURE S2 Flow cytometric analysis of MLN DC subsets. **(A)** The gating strategy used to identify MLN DC subsets is shown. **(B)** RALDH activity in MLN DC subsets from young BALB/c mice was analyzed by flow cytometry. RALDH activity of each MLN DC subset was measured as fluorescent intensity of ALDEFLUOR reagent and shown as histograms (dashed line: ALDEFLUOR + DEAB) or mean fluorescent intensity (MFI). DEAB (ALDH inhibitor) was used to confirm the RALDH activity. Data are shown as the mean of RALDH activity in pooled cells from multiple mice (*n* = 12).

FIGURE S3 Gating strategy used to identify CD4^+^Foxp3^+^ cells in a DC-T cells coculture system. Single cells were gated, and autofluorescence^+^ cells (PerCP-Cy5.5 was measured as autofluorescence) were depleted from FSC-SSC gated cells. OVAp-specific (D10 TCR^+^) CD4^+^ T cells were gated, and the proportion of Foxp3^+^CD4^+^ cells were analyzed.

FIGURE S4 Gating strategy used to identify CD4^+^Foxp3^+^ cells in an RA administration experiment. Single cells were gated, and autofluorescence^+^ cells (FITC was measured as autofluorescence) were depleted from FSC-SSC gated cells. CD4^+^ T cells were gated, and the proportion of Foxp3^+^CD4^+^ cells were analyzed.

FIGURE S5 Impact of aging on T cells expressing Foxp3 in a DC-T cell coculture system. **(A)** The gating strategy used to identify CD4^+^Foxp3^+^ cells is shown. **(B)** MLN DCs (2 × 10^4^ cells) from BALB/c mice and splenic CD4^+^ cells (2 × 10^5^ cells) from RAG2KO/DO11.10 mice were cocultured in the presence of OVAp (100 nM) and human TGF-β (2 ng/ml) (purchased from R&D systems, Minneapolis, MN, USA) in a 96-well flat bottom plate (Corning) in 200 μl of 10% FCS-RPMI for 72 hours in a 5% CO_2_ humidified atmosphere at 37°C. The proportion of Foxp3^+^ cells in CD4^+^ cells after coculture was analyzed. Data are shown as the mean±SD of independent wells (*n* = 3) and each point indicates the data from independent wells. The plot shows one representative dataset of two independent experiments. Turkey’s HSD test was used for statistical analysis. Values not sharing a common letter are significantly different (*p* < 0.05).

FIGURE S6 Impact of aging on T cells expressing CCR9 in a DC-T cell coculture system. **(A)** The gating strategy used to identify CD4^+^CCR9^+^ cells is shown. Anti-CCR9-FITC (CW-1.2) was purchased from BioLegend. **(B)** MLN DCs (2 × 10^4^ cells) from BALB/c mice and splenic CD4^+^ cells (2 × 10^5^ cells) from RAG2KO/DO11.10 mice were cocultured in the presence of OVAp (100 nM) and RA (1 μM) (FUJIFILM Wako Pure Chemical Corporation) in a 96-well flat bottom plate (Corning) in 200 μl of 10% FCS-RPMI for 72 hours in a 5% CO_2_ humidified atmosphere at 37°C. The proportion of CCR9^+^ cells in CD4^+^ cells after coculture was analyzed. Data are shown as the mean±SD of independent wells (*n* = 3) and each point indicates the data from independent wells. The plot shows one representative dataset of two independent experiments. Turkey’s HSD test was used for statistical analysis. Values not sharing a common letter are significantly different (*p* < 0.05).
