# Supplementary figures and images for: Age-Dependent Decrease in the Induction of Regulatory T Cells Is Associated With Decreased Expression of RALDH2 in Mesenteric Lymph Node Dendritic Cells
Source: Front Immunol. 2020 Aug 11;11:1555. doi: 10.3389/fimmu.2020.01555 (PMC7432217; doi:10.3389/fimmu.2020.01555)

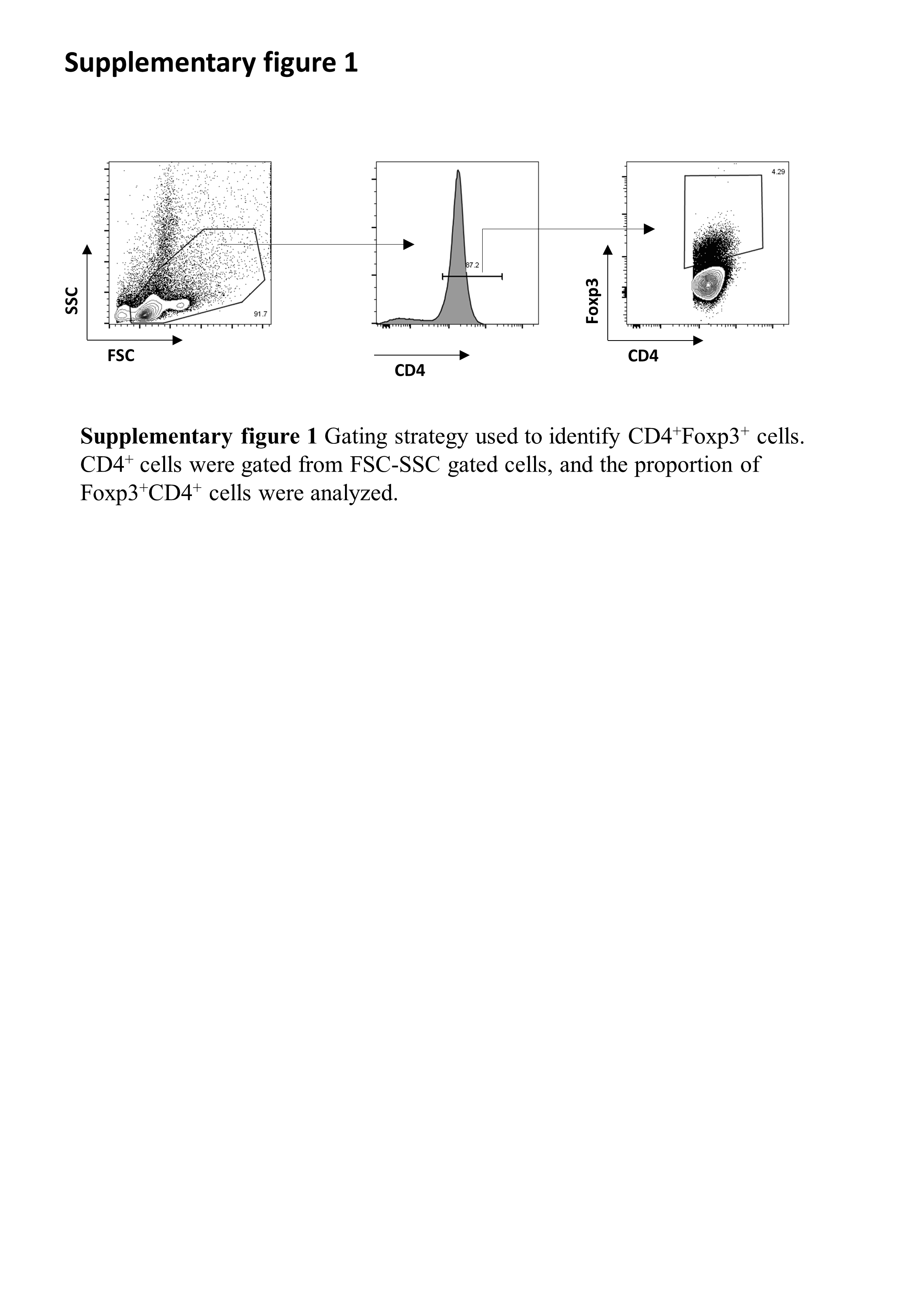

Supplement: FIGURE S1 — Gating strategy used to identify CD4+Foxp3+ cells. CD4+ cells were gated from FSC-SSC gated cells, and the proportion of Foxp3+CD4+ cells were analyzed. [file Image_1.TIF]

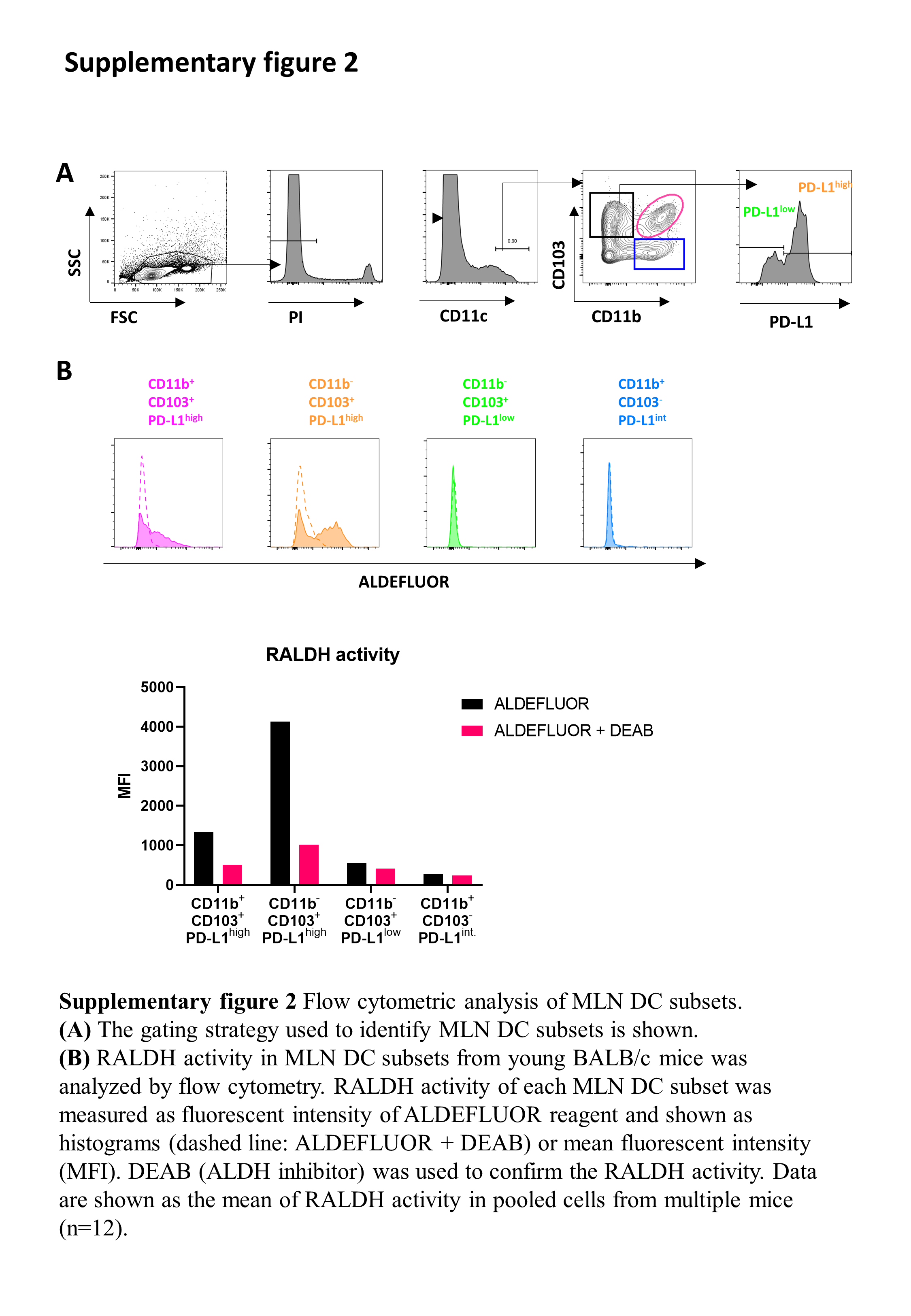

Supplement: FIGURE S2 — Flow cytometric analysis of MLN DC subsets. (A) The gating strategy used to identify MLN DC subsets is shown. (B) RALDH activity in MLN DC subsets from young BALB/c mice was analyzed by flow cytometry. RALDH activity of each MLN DC subset was measured as fluorescent intensity of ALDEFLUOR reagent and shown as histograms (dashed line: ALDEFLUOR + DEAB) or mean fluorescent intensity (MFI). DEAB (ALDH inhibitor) was used to confirm the RALDH activity. Data are shown as the mean of RALDH activity in pooled cells from multiple mice (n = 12). [file Image_2.TIF]

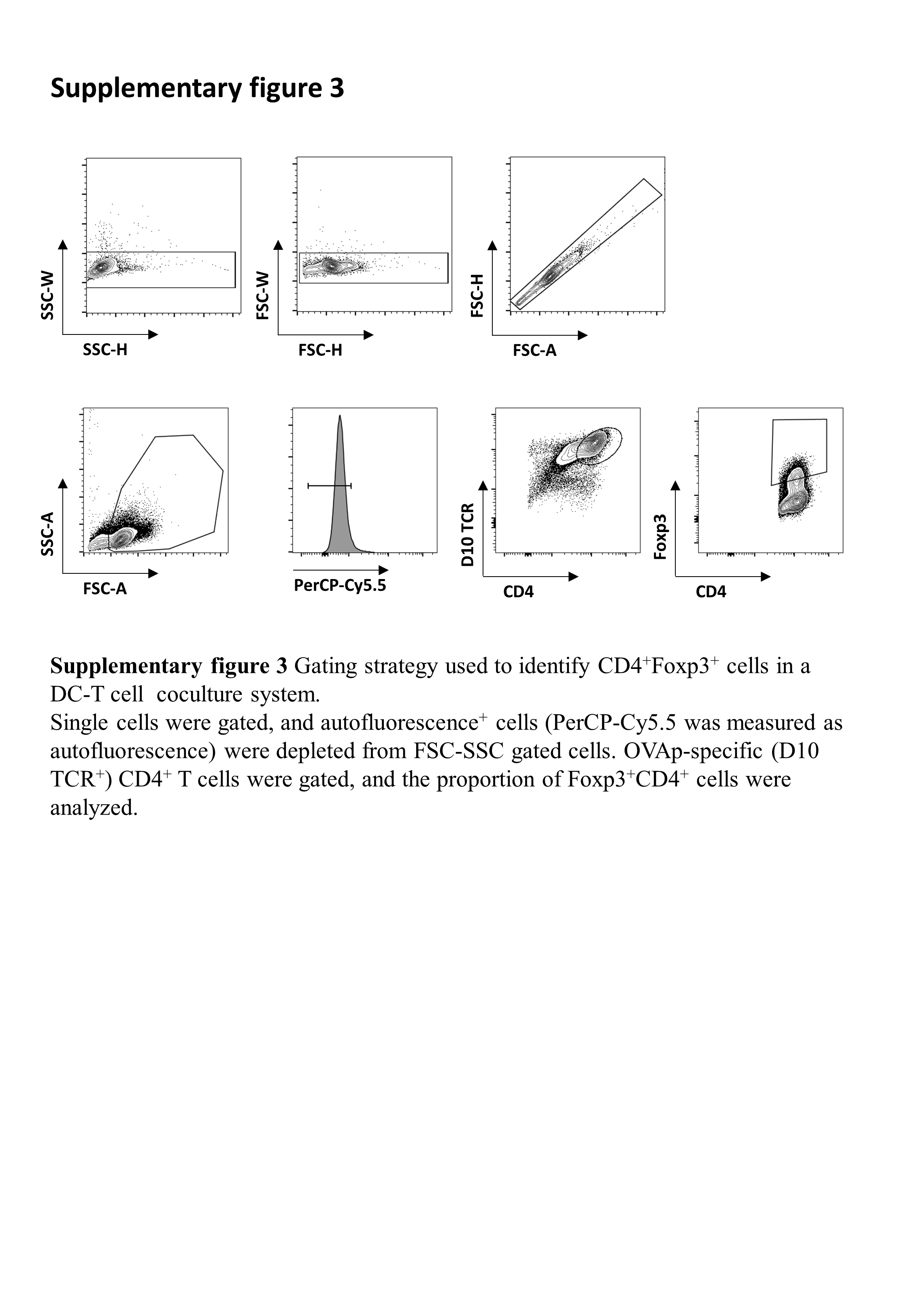

Supplement: FIGURE S3 — Gating strategy used to identify CD4+Foxp3+ cells in a DC-T cells coculture system. Single cells were gated, and autofluorescence+ cells (PerCP-Cy5.5 was measured as autofluorescence) were depleted from FSC-SSC gated cells. OVAp-specific (D10 TCR+) CD4+ T cells were gated, and the proportion of Foxp3+CD4+ cells were analyzed. [file Image_3.tif]

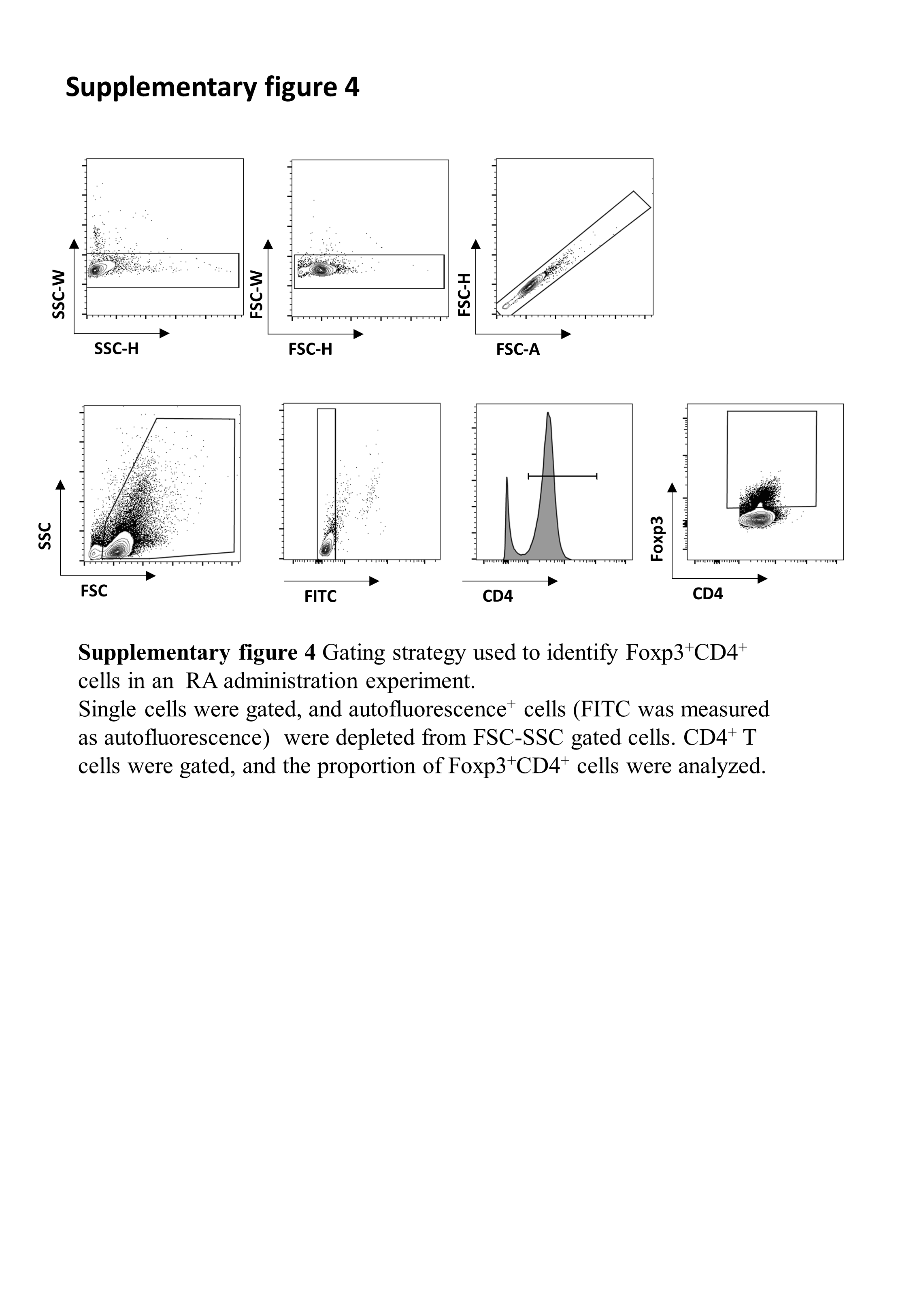

Supplement: FIGURE S4 — Gating strategy used to identify CD4+Foxp3+ cells in an RA administration experiment. Single cells were gated, and autofluorescence+ cells (FITC was measured as autofluorescence) were depleted from FSC-SSC gated cells. CD4+ T cells were gated, and the proportion of Foxp3+CD4+ cells were analyzed. [file Image_4.tif]

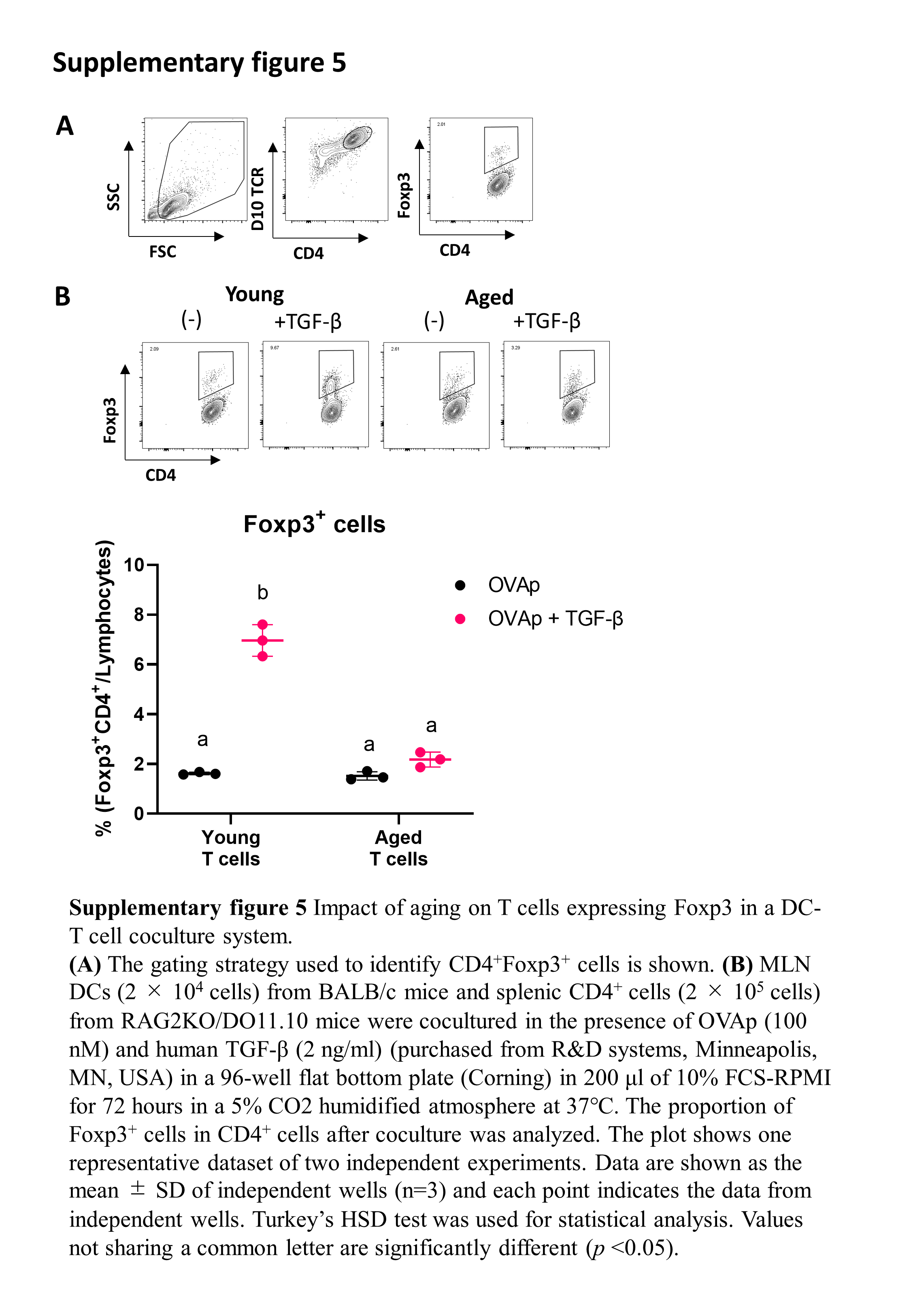

Supplement: FIGURE S5 — Impact of aging on T cells expressing Foxp3 in a DC-T cell coculture system. (A) The gating strategy used to identify CD4+Foxp3+ cells is shown. (B) MLN DCs (2 × 10^4^ cells) from BALB/c mice and splenic CD4+ cells (2 × 10^5^ cells) from RAG2KO/DO11.10 mice were cocultured in the presence of OVAp (100 nM) and human TGF-β (2 ng/ml) (purchased from R&D systems, Minneapolis, MN, USA) in a 96-well flat bottom plate (Corning) in 200 μl of 10% FCS-RPMI for 72 hours in a 5% CO2 humidified atmosphere at 37°C. The proportion of Foxp3+ cells in CD4+ cells after coculture was analyzed. Data are shown as the mean±SD of independent wells (n = 3) and each point indicates the data from independent wells. The plot shows one representative dataset of two independent experiments. Turkey’s HSD test was used for statistical analysis. Values not sharing a common letter are significantly different (p < 0.05). [file Image_5.TIF]

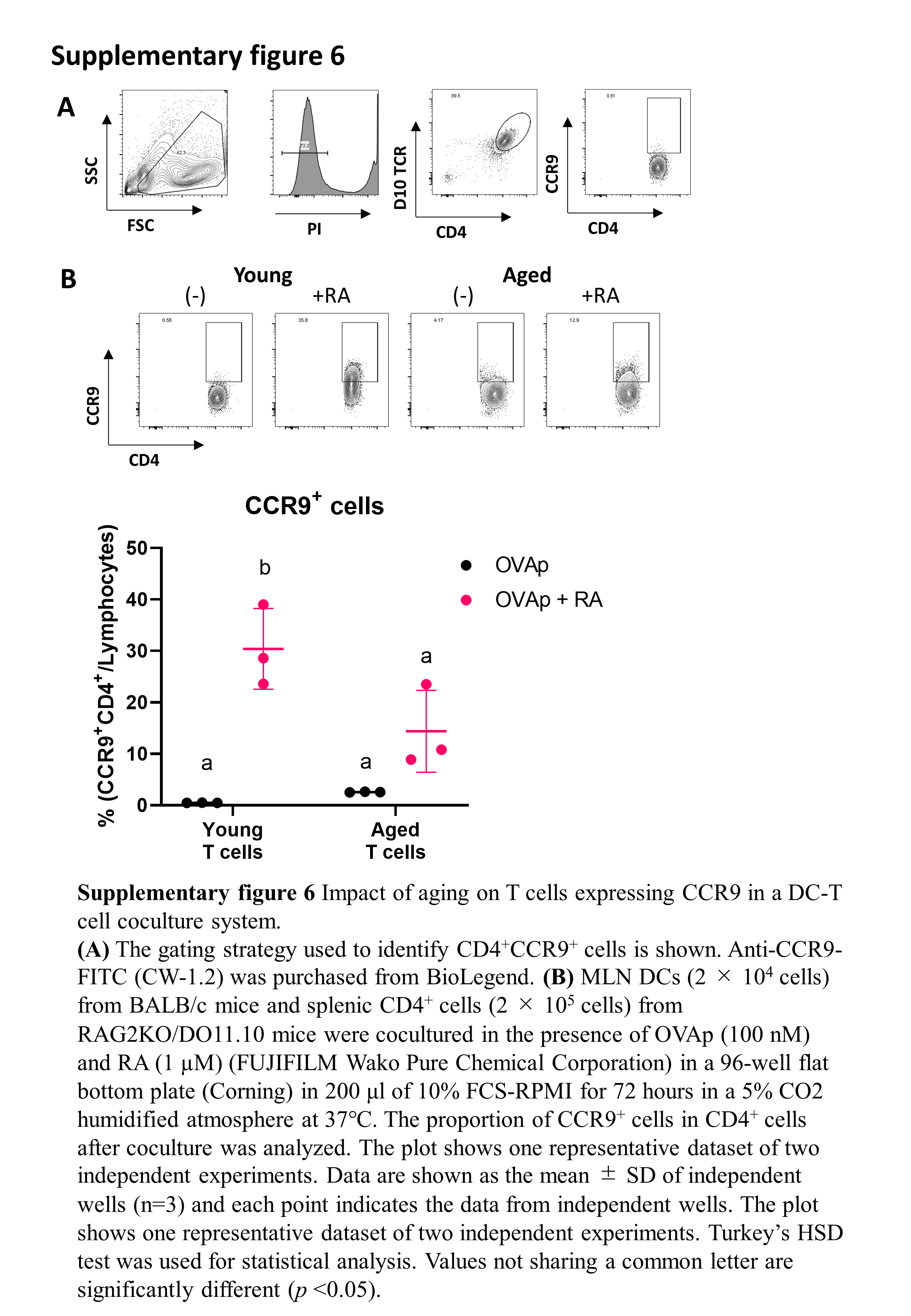

Supplement: FIGURE S6 — Impact of aging on T cells expressing CCR9 in a DC-T cell coculture system. (A) The gating strategy used to identify CD4+CCR9+ cells is shown. Anti-CCR9-FITC (CW-1.2) was purchased from BioLegend. (B) MLN DCs (2 × 10^4^ cells) from BALB/c mice and splenic CD4+ cells (2 × 10^5^ cells) from RAG2KO/DO11.10 mice were cocultured in the presence of OVAp (100 nM) and RA (1 μM) (FUJIFILM Wako Pure Chemical Corporation) in a 96-well flat bottom plate (Corning) in 200 μl of 10% FCS-RPMI for 72 hours in a 5% CO2 humidified atmosphere at 37°C. The proportion of CCR9+ cells in CD4+ cells after coculture was analyzed. Data are shown as the mean±SD of independent wells (n = 3) and each point indicates the data from independent wells. The plot shows one representative dataset of two independent experiments. Turkey’s HSD test was used for statistical analysis. Values not sharing a common letter are significantly different (p < 0.05). [file Image_6.TIF]
